# Sex differences in event-related potentials and attentional biases to emotional facial stimuli

**DOI:** 10.3389/fpsyg.2014.01477

**Published:** 2014-12-22

**Authors:** Daniela M. Pfabigan, Elisabeth Lamplmayr-Kragl, Nina M. Pintzinger, Uta Sailer, Ulrich S. Tran

**Affiliations:** ^1^Social, Cognitive and Affective Neuroscience Unit, Department of Basic Psychological Research and Research Methods, Faculty of Psychology, University of Vienna, Vienna, Austria; ^2^Department of Applied Psychology: Health, Development, Enhancement and Intervention, Faculty of Psychology, University of Vienna, Vienna, Austria; ^3^Department of Psychology, Faculty of Social Sciences, University of Gothenburg, Gothenburg, Sweden; ^4^Department of Basic Psychological Research and Research Methods, Faculty of Psychology, University of Vienna, Vienna, Austria

**Keywords:** sex differences, dot probe paradigm, attentional bias, difference wave approach, probe P1 amplitudes, alexithymia

## Abstract

Attentional processes play an important role in the processing of emotional information. Previous research reported attentional biases during stimulus processing in anxiety and depression. However, sex differences in the processing of emotional stimuli and higher prevalence rates of anxiety disorders among women, compared to men, suggest that attentional biases may also differ between the two sexes. The present study used a modified version of the dot probe task with happy, angry, and neutral facial stimuli to investigate the time course of attentional biases in healthy volunteers. Moreover, associations of attentional biases with alexithymia were examined on the behavioral and physiological level. Event-related potentials were measured while 21 participants (11 women) performed the task, utilizing also for the first time a difference wave approach in the analysis to highlight emotion-specific aspects. Women showed overall enhanced probe P1 amplitudes compared to men, in particular after rewarding facial stimuli. Using the difference wave approach, probe P1 amplitudes appeared specifically enhanced with regard to congruently presented happy facial stimuli among women, compared to men. Both methods yielded enhanced probe P1 amplitudes after presentation of the emotional stimulus in the left compared to the right visual hemifield. Probe P1 amplitudes correlated negatively with self-reported alexithymia, most of these correlations were only observable in women. Our results suggest that women orient their attention to a greater extent to facial stimuli than men and corroborate that alexithymia is a correlate of reduced emotional reactivity on a neuronal level. We recommend using a difference wave approach when addressing attentional processes of orientation and disengagement also in future studies.

## INTRODUCTION

Attentional processes play an important role in the processing of emotional information and with regard to the development and maintenance of symptoms of anxiety and depression: anxious and depressive subjects allocate more attention to threatening stimuli and less attention to pleasant stimuli and cues of reward (e.g., Bar-Haim et al., 2007; Frewen et al., 2008; Staugaard, 2010; Yiend, 2010). Women are known to have higher prevalence rates of anxiety disorders than men (e.g., Kessler et al., 2005) and it is also an established finding that there are sex differences in the processing of emotional stimuli (Cahill, 2006). Killgore and Yurgelun-Todd (2001) observed enhanced activation in the right amygdala when presenting happy faces only in their male participants. In contrast, the presentation of fearful faces evoked enhanced left amygdala activation in both sexes. Derntl et al. (2009) also reported subtle sex differences in amygdala activation. Enhanced bilateral amygdala activation was positively related to better fear recognition only in their male participants. Importantly, the amygdala is considered to be actively involved in driving emotional enhancement, i.e., exerting modulatory influence on visual processing of emotional stimuli (Vuilleumier et al., 2004).

Based on the enhanced prevalence rates for anxiety disorders in women and reported sex differences during the processing of emotional stimuli, it can be hypothesized that also attentional biases differ between the two sexes. There is indeed a growing number of attentional bias studies that reported sex differences on the behavioral level (Tan et al., 2011; Donges et al., 2012; Tran et al., 2013), but also on the neuronal level (Sass et al., 2010). Women have a greater ability than men to perceive and respond to positive stimuli at an automatic processing level (Donges et al., 2012), and show enhanced neural activity during early visual processing stages compared to men, regardless of the emotional content of the stimuli (Sass et al., 2010). Moreover, in anxiety, attentional biases toward threat may be a phenomenon that is limited to women as suggested by recent studies (Tan et al., 2011; Tran et al., 2013).

A widely used paradigm to investigate attentional biases is the dot probe task (see Bar-Haim et al., 2007). In the original version (MacLeod et al., 1986), participants were shortly presented with two words on the left and right side of a computer screen. One of these words was emotionally valenced, the other one was neutral. Immediately after display offset, a dot (i.e., the “probe”) appeared in the location of one of the words; either in the location of the emotional stimulus (consequently a “congruent trial”) or in the position of the neutral one (consequently an “incongruent trial”). Participants’ task was to indicate visual detection of the probe by pressing a corresponding button as fast as possible. Following theoretical considerations (MacLeod et al., 1986), response times should be shorter for congruent trials in case attention is captured by the emotional stimulus. If attention is directed away from the emotional stimulus (i.e., an avoidance reaction), response times should be shorter for incongruent trials. Subtracting mean response times in congruent and incongruent trials results in a commonly used bias index (BI). This BI is positive when attention is drawn to emotional stimuli and negative when emotional stimuli are avoided. However, this BI might not be able to distinguish different attentional processes from each other. Positive scores may either be due to fast reactions in congruent trials (suggesting increased attention toward target stimuli) and/or due to slow reactions in incongruent trials (suggesting delayed disengagement from target stimuli; Salemink et al., 2007; Cisler and Koster, 2010). To avoid this ambiguous constellation, adding trials with two neutral stimuli may allow to differentiate fast orienting more clearly from a difficulty to disengage as reaction times in the neutral–neutral trials may serve as a baseline measure. Studies applying these modified task version suggested that anxiety-related attentional biases seem to reflect specifically effects of delayed disengagement, but not of increased attention (Cisler and Koster, 2010).

Reaction times are one dependent measure to assess attentional biases, but may be prone to measurement error (e.g., Waechter et al., 2014). To further elucidate attentional processes, previous studies have also investigated physiological measures during the dot probe task. Event-related potentials (ERPs) evoked by visual task displays of the dot probe task are a useful tool to further disentangle underlying attentional processes since they provide millisecond precision. The current study particularly focused on an ERP evoked by the probe presentation—the P1 component (termed “probe P1” in the following)—as did several other attentional bias studies (see below).

The P1 is a positive-going ERP with peak latencies between 100 and 130 ms after visual presentation at parieto-occipital and occipital electrode positions (Luck, 2005). It indexes an early stage of visual processing, as with regard to luminance or contrast (i.e., low-level visual features; Luck, 2005). However, apart from low-level visual processing, the P1 amplitude is also modulated by top-town attentional processes. P1 amplitudes were reported to be enhanced for attended, compared to unattended, stimuli in spatial attention paradigms (Hillyard and Anllo-Vento, 1998; Luck and Ford, 1998) and have also been linked to emotional face categorization processes (Linkenkaer-Hansen et al., 1998; Pizzagalli et al., 2002). Specifically, modulation of P1 amplitudes is larger for negative emotional faces than for positive emotional faces (Ito et al., 1998; Smith et al., 2003).

Recent studies in healthy participants reported enhanced probe P1 amplitudes after angry compared to happy faces (Santesso et al., 2008) and after fearful compared to happy faces (Pourtois et al., 2004) in a dot probe paradigm, and enhanced P1 amplitudes to emotionally congruently primed targets (i.e., fearful faces) in contrast to incongruently primed ones in a spatial cueing paradigm (Brosch et al., 2011). Santesso et al. (2008) interpreted their results as indicative of increased sensory gating for emotionally cued stimuli in the visual cortex and in line with theories on hyper vigilance toward threat. However, in their tasks, Santesso et al. (2008) and Pourtois et al. (2004) were not able to distinguish increased vigilance from disengagement difficulties. Brown et al. (2010) specifically reported that both evolutionary relevant (e.g., pictures of snakes or spiders) and irrelevant threatening stimuli (e.g., pictures of knives and syringes) evoke enhanced probe P1 amplitudes in congruently primed trials, compared to incongruently primed ones. This finding speaks for the universality of the so-called threat-superiority effect—meaning that any threatening stimuli accompanied by fear or danger easily capture attention compared to non-threatening ones (Öhman et al., 2001; Smith et al., 2003; Blanchette, 2006). In contrast, Eldar et al. (2010) found no probe P1 amplitude variation in response to angry or happy faces in anxious and non-anxious participants, using a block design to present the different emotions.

In summary, the results of extant studies on probe P1 amplitudes are rather inconsistent. Notably, participant sex was not controlled for in these studies and most of them did not include neutral stimuli in their paradigms (but see Eldar et al., 2010, and O’Toole and Dennis, 2012) or did not analyze them. The internal validity and generalizability of previous studies thus appears, both on the behavioral and the physiological level, limited. For the ERPs, neutral trials allow the calculation of differences waves to further extract relevant ERP amplitude variation. Neutral–neutral stimulus pairs could thus serve as individual baselines when calculating participant- and emotion-wise difference waves. The difference wave approach might be better suited to disentangle vigilance and disengagement effects (Luck, 2005).

In order to elucidate and to expand on previous inconsistent findings, the present study focused on the examination of sex differences in probe P1 amplitudes, utilizing the dot probe paradigm with emotional facial stimuli. As a novel and unique procedure in neuroscientific attentional bias research, we implemented a difference wave approach for the study of attentional biases. This was possible because we included neutral–neutral trials in our electroencephalogram (EEG) paradigm, as suggested by Salemink et al. (2007) for behavioral data. Given previous results on sex differences with regard to attentional processes and attentional biases (see above), we expected overall enhanced ERP amplitudes among women compared to men. As we expected ERP amplitudes to be specifically enhanced during early stimulus processing stages for attended stimuli (Luck, 2005), the P1 component time interval lay in the focus of the present study. Data of a community sample were used, as we were interested in sex differences in the general population. Psychological symptoms were assessed in the course of data acquisition. Potential sex effects were explored both in behavioral and neuronal correlates of the dot probe task, using alternative bias indices as proposed by Salemink et al. (2007) to differentiate fast orienting from a difficulty to disengage on the behavioral level (see Materials and Methods) and a difference wave approach for ERPs. Additionally, we assessed associations of attentional biases on the behavioral and physiological level with alexithymia, which has been repeatedly reported to be related to emotion processing (e.g., Franz et al., 2004; Eichmann et al., 2008; Reker et al., 2010). Alexithymia can be described as the inability to identify, describe, regulate, and express emotions (Sifneos, 1976) and is considered to be a continuous personality trait (Jessimer and Markham, 1997). Previous research has linked disturbed emotion regulation in alexithymia with deficits in the processing stream of emotional stimuli (Lane et al., 2000; Berthoz et al., 2002; Mantani et al., 2005). This relation might be also seen in attentional biases. Moreover, several studies reported P1 amplitude variation in relation to alexithymia when participants were presented with emotional stimuli (Schaefer et al., 2007; Pollatos and Gramann, 2011). Therefore, we assessed alexithymic traits in the participants of the current dot probe experiment to assess a possible link between attentional biases and alexithymia.

## MATERIALS AND METHODS

### PARTICIPANTS

Twenty-one volunteers (11 women; all sampled from the community) participated in the present study. Mean age of all participants was 27.3 ± 3.58 years, ranging from 23 to 34. All participants were right-handed (Edinburgh Handedness Inventory; Oldfield, 1971), had normal or corrected-to-normal vision, and reported no past or present neurological or psychiatric disorder. Most participants (*n* = 20; 95%) reported non-clinical levels of current psychological symptoms [*T* scores <63 in the relevant scales of the SCL-90-R (Symptom Checklist-90-Revised); see below]. One male participant (5%) reported elevated levels in anxiety, depression, and global psychological distress. This was not unexpected as using a cutoff of 63, roughly 10% of the general population are expected to show elevated scores.

This study was conducted in accordance with the Declaration of Helsinki (1983) and local guidelines of the University of Vienna and the Faculty of Psychology. All participants gave written informed consent prior to the experiment.

### QUESTIONNAIRES

Prior to the EEG data collection, participants completed several psychological questionnaires.

#### Psychological symptoms

Current psychological distress, depression, and anxiety were assessed with the 90-item Symptom Checklist (SCL-90-R; German version: Franke, 2002). The SCL-90-R assesses the prevalence and distress caused by a variety of symptoms during the last 7 days. Depression and anxiety were assessed with 13 and 10 items, respectively. Psychological distress (Global Severity Index; GSI) is operationalized as the mean of all 90 items. Items were scored from 0 (*not at all*) to 4 (*extremely*). In the current sample, Cronbach alpha for scales of depression, anxiety, and psychological distress was 0.81, 0.85, and 0.95, respectively. *T* scores ≥63 may be considered clinically relevant, according to the published norm data of the instrument.

#### Self-reported alexithymia

Ratings of alexithymia were obtained with the 26-item Toronto Alexithymia Scale (TAS-26; German version: Kupfer et al., 2001). The TAS-26 assesses three components of alexithymia: difficulties in the identification of feelings (DIF; seven items), difficulties describing feelings (DDF; five items), and externally oriented thinking (EOT; six items). Items were scored from 1 (*strongly disagree*) to 5 (*strongly agree*). Cronbach alpha in the current sample was 0.68 (DIF), 0.61 (DDF), and 0.33 (EOT), which is in accordance with published validity data, except for EOT, where Cronbach alpha appeared unacceptably low in the current sample. The TAS-26 also allows the computation of a total score (Cronbach alpha = 0.64 in the current sample) that was, however, not used in the present study.

### TASK AND PROCEDURE

The synchronization of the stimulus presentation with the EEG recording was implemented by E-Prime 2.0 software (Psychology Software Tools, Inc., Sharpsburg, PA, USA) running on a Pentium IV, 3.00 GHz machine. During EEG data collection, participants were seated comfortably in a sound-attenuated room in front of a 21 inch cathode ray tube monitor (Sony GDM-F520; 75 Hz refresh rate) with approximately 70 cm distance to the screen. A modified version of the dot probe paradigm by MacLeod et al. (1986) was applied. Participants’ task was to indicate the location of a probe stimulus on the screen via corresponding button press. Each trial started with the central presentation of a black fixation cross against a white background for 750 ms. Subsequently, two pictures depicting faces were presented to the left and to the right of the fixation cross (i.e., left or right visual hemifield; picture size: 4 cm × 5 cm; distance from fixation cross to picture center: 4 cm). These pictures were taken from the FACES database (Ebner et al., 2010), utilizing emotional and neutral facial expressions of four female posers and four male posers, and presented for 500 ms. Afterward, the faces disappeared and a black dot (the “probe”) was blended in for at most 3000 ms, either on the position of the left or the right face picture. Participants had to indicate dot location, i.e., right or left half of the screen, by pressing a corresponding button on a standard keyboard with their right (“j”) or left (“f”) index finger. Immediately after the button press, the dot disappeared and the fixation cross was presented again with a variable duration of 750–1000 ms. Each trial consisted either of the combination of an emotional and a neutral face picture by the same poser or of the combination of two neutral face picture by the same poser. Emotional facial expressions depicted anger, disgust, fear, happiness, and sadness (Ekman, 1992). Each emotion-neutral pair was presented twelve times per poser, the location of the emotional face picture and the location of the subsequent dot were counterbalanced across trials. Each neutral–neutral face pair was presented six times per poser. Overall, the experiment consisted of 528 trials. Emotional and neutral pairs were presented randomly. Prior to the experiment, participants completed 16 training trials with neutral–neutral pairs with eight different posers (four female, four male posers) to get familiar with the experimental paradigm. Concerning emotional-neutral pairs, congruent trials were defined as trials where the dot replaced an emotional face, whereas trials where the dot replaced the neutral facial expression were considered as incongruent trials. For the neutral–neutral face pairs, no congruency effect was assessable. Thus, each dot replacement was considered as neutral. After blocks of 44 trials, participants were given short breaks if needed. Overall, EEG data collection took around 45 minutes.

### DATA ACQUISITION

Electroencephalogram was recorded from 59 Ag/AgCl ring electrodes which were embedded in a fabric electrode cap in an equidistant fashion (EASYCAP GmbH, Herrsching, Germany; model M10). A further four electrodes were placed at both outer canthi and 1 cm above and below the left eye to record horizontal and vertical electrooculogram (EOG). These bipolar EOG recordings were used off-line for eye-movement correction. Electrodes on the seventh vertebra and on the right sterno-clavicular joint served as reference site (Stephenson and Gibbs, 1951). Subsequently, a skin-scratching procedure was applied to each electrode site to keep electrode impedances below 2 kΩ (Picton and Hillyard, 1972). EEG signals were amplified using an AC amplifier set-up with a time constant of 10 s (Ing. Kurt Zickler GmbH, Pfaffstätten, Austria). EEG was recorded within a frequency range of 0.016–125 Hz and sampled at 250 Hz for digital storage.

### DATA ANALYSIS

As prior evidence (Tran et al., 2013) revealed strongest effects for happy and angry faces among both men and women, only happy, angry, and neutral face pairs were considered for analysis in the present study.

#### Behavioral data analysis

Response times were defined as the interval from dot onset to button press. Trials with response times faster than 200 ms and slower than 1000 ms were discarded. Only correct trials were considered for analysis (less than 1.5% of all trials were incorrect). Mean reaction times were assessed and used to calculate emotion-wise bias indices. First, the commonly used BI was calculated via subtracting mean response times of congruent trials from mean response times of incongruent trials. Secondly, the orienting index (OI) was calculated via subtracting the mean response times of congruent trials from mean response times of neutral trials. Thirdly, the disengaging index (DI) was calculated via subtracting mean response times of neutral trials from mean response times of incongruent trials. OI and DI, as proposed by Salemink et al. (2007), are considered to disentangle processes of increased and facilitated orientation toward target stimuli (i.e., OI) from processes of delayed and decreased disengagement from target stimuli (i.e., DI). Previous research indicated that OI and DI scores may be more valid indicators of attentional biases than the commonly applied BI scores (Tran et al., 2013). Note that the index scores OI and DI add up to the BI, but depend on neutral–neutral trials for computation. The presence of attentional biases was assessed using one-sample *t*-tests versus 0 in the overall sample, as well as among women and men separately. Moreover, we tested whether or not sex differences were also observable in reaction times and questionnaire data.

#### EEG data analysis

Prior to data analysis, participant- and channel-specific coefficients were calculated for weighting vertical and horizontal eye movements which were assessed during two calibration trials administered prior to the experiment. Subsequently, these weighted EOG signals were subtracted from experimental EEG data (Bauer and Lauber, 1979). Off-line data analysis was carried out using EEGLAB 6.0.3b (Delorme and Makeig, 2004) with Matlab 7.9.0 (The MathWorks, Inc., Natick, MA, USA). A low-pass filter with a cut-off frequency of 30 Hz (roll-off 6 dB/octave) was applied to the data. Data were epoched starting 100 ms prior to dot presentation with each epoch lasting 500 ms. The mean of the first 100 ms served as baseline interval. Data were epoched according to emotion (anger, happy, neutral), congruency condition (congruent, incongruent) and according to the hemifield in which the emotional face was presented prior to dot onset (right, left hemifield). The factors congruency and hemifield was only available for angry and happy faces. Nine experimental conditions were derived after averaging participant- and condition-wise: *anger-congruent-R*, *anger-congruent-L anger-incongruent-R*, *anger-incongruent-L*, *happy-congruent-R*, *happy-congruent-L*, *happy-incongruent-R*, *happy-incongruent-L*, and *neutral*.

A semi-automatic artifact removal procedure was applied to these epochs. Artifact-afflicted trials with voltage values exceeding ±70 *μ*V or with voltage drifts of more than 50 *μ*V were automatically marked by EEGLAB. During subsequent visual inspection, the automatic markings were controlled and artifact-afflicted trials were discarded from further analysis. Mean amplitudes were assessed for probe P1 amplitudes (interval: 80–120 ms) at midline electrode location Oz for all conditions.

Probe P1 mean amplitudes were investigated with a linear mixed model, examining the factors sex, emotion (anger vs. happy vs. neutral), congruency (incongruent vs. congruent), and hemifield (right vs. left); congruency and hemifield were both nested within emotion (effects of congruency applied only to the emotions anger and happy, but not neutral; the same was also true with regard to hemifield). Such a doubly nested design may not be directly investigated with classical ANOVA, but demands utilization of specific analysis tools, like the linear mixed model. Parameters in the linear mixed model were estimated with maximum likelihood, using an unstructured covariance matrix. In addition to the results of the effect tests, we report here Cohen’s *d* of significant effects, derived from the effect estimates of the fitted model, as no direct estimates of explained variance are provided in linear mixed models.

Additionally, difference waves were calculated with the artifact-corrected EEG data, yielding measures on a physiological level that were comparable to Salemink et al.’s (2007) bias indices on the behavioral level. However, in order to account for the nature of the ERP data, neutral trials always served as subtrahend in our calculations to allow direct comparison of ERP amplitude variation. Eight experimental conditions were derived: *anger-congruent-R-diff*, *anger-congruent-L-diff*, *anger-incongruent-R-diff*, *anger-incongruent-L-diff*, *happy-congruent-R-diff*, *happy-congruent-L-diff*, *happy-incongruent-R-diff*, and *happy-incongruent-L-diff*. Mean amplitudes for probe P1 amplitudes were also extracted at Oz, 80–120 ms after probe onset. These probe P1 mean difference wave amplitudes were subjected to a four-way mixed-model ANOVA with the between-subject factor sex, and the within-subject factors emotion (anger vs. happiness), congruency (incongruent vs. congruent), and hemifield (right vs. left). Classical ANOVA could be utilized here, as all factors were fully crossed with one another (balanced design). Significant interaction effects in the ANOVA were explored with *t*-tests.

Furthermore, Spearman correlations (*r*_s_) were calculated to explore the associations between probe P1 and probe P1 difference wave amplitude variations, behavioral measures, and questionnaire data. Significance was set at *p* < 0.05 (two-sided) for all tests; *p* < 0.10 was interpreted as borderline significant. Partial eta-squared (${\text{η}}_{\text{p}}^{\text{2}}$) and Cohen’s *d* are reported as effect sizes, values of ${\text{η}}_{\text{p}}^{\text{2}}$ = 0.01/*d* = 0.20, ${\text{η}}_{\text{p}}^{\text{2}}$ = 0.06/*d* = 0.50, and ${\text{η}}_{\text{p}}^{\text{2}}$ = 0.14/*d* = 0.80 representing small, medium, and large effects. Fisher’s *z*-test was applied to assess significant differences in correlation coefficients. Statistical analysis was performed using PASW 18 (SPSS Inc., IBM Corporation, NY, USA).

## RESULTS

### BEHAVIORAL DATA

Table 1 provides descriptive statistics on all bias indices and psychological measures. The overall sample showed BI scores for happy faces that were significantly lower than 0 [*t*(20) = –4.49, *p* < 0.001, *d* = –0.98], indicating avoidance of happy faces following the classical BI interpretation (MacLeod et al., 1986). Taking the neutral trials into account, this result was reflected in the borderline significance of OI happy scores [*t*(20) = 1.74, *p* = 0.097, *d* = 0.38] which rather speaks for increased orienting toward happy faces (Salemink et al., 2007; Cisler and Koster, 2010). No other BI reached the significance level (all *p*-values ≥0.280). When splitting the sample in male and female subgroups, BI scores for happy faces were still significantly lower than 0 in the male group [*t*(9) = –4.92, *p* = 0.001, *d* = –1.56], and borderline significant in the female group [*t*(10) = –2.14, *p* = 0.058, *d* = –0.65]. No other BI reached significance in the subgroups (all *p*-values ≥0.139). Using a more stringent significance level of *p* < 0.01 to control for multiple testing, only BI scores for happy faces in the overall sample and among men reached significance. *Per se*, bias indices did not differ between women and men (all *p*-values ≥0.128).

**Table 1:** Means and standard deviations of bias index scores, reaction times, and psychological measures.

	**Total sample**	**SD**	**Men (*n* = 10)**	**SD**	**Women (*n* = 11)**	**SD**	**Statistics sex differences**
**Bias indices**							
BI anger	2.17	9.10	4.39	9.14	0.16	9.00	*t*(19) = –1.07, *p* = 0.300
BI happy	–5.35***	7.80	–8.08***	7.92	–2.86^+^	7.13	*t*(19) = 1.59, *p* = 0.128
OI anger	2.44	11.63	4.82	14.76	0.27	7.97	*t*(19) = –0.89, *p* = 0.348
OI happy	–3.31^+^	13.00	–3.39	16.37	–3.23	9.83	*t*(19) = 0.03, *p* = 0.978
DI anger	–0.26	8.80	–0.44	8.37	–0.11	9.58	*t*(19) = 0.08, *p* = 0.935
DI happy	–2.04	10.97	–4.69	11.70	0.37	10.19	*t*(19) = 1.06, *p* = 0.303
**Reaction times**							
Anger congruent	359.00	34.81	369.01	44.17	349.90	21.85	*t*(19) = –1.28, *p* = 0.218
Anger incongruent	361.17	37.88	373.39	45.70	350.07	26.56	*t*(19) = –1.45, *p* = 0.164
Happy congruent	364.75	36.29	377.22	44.23	353.41	23.99	*t*(19) = –1.55, *p* = 0.137
Happy incongruent	359.40	36.23	369.14	45.01	350.55	24.91	*t*(19) = –1.19, *p* = 0.250
Neutral	361.44	37.28	373.83	47.29	350.17	21.77	*t*(12.4) = –1.45, *p* = 0.172
**SCL-90**							
Depression	4.71	5.25	4.00	6.83	5.36	3.50	*t*(19) = 0.58, *p* = 0.566
Anxiety	3.05	3.81	2.90	4.15	3.18	3.68	*t*(19) = 0.17, *p* = 0.871
GSI	0.27	0.25	0.24	0.32	0.29	0.18	*t*(19) = 0.44, *p* = 0.663
**TAS-26**							
DIF	11.00	2.77	11.00	2.31	11.00	3.26	*t*(19) < 0.01, *p* > 0.999
DDF	10.76	2.70	12.40	2.07	9.27	2.37	*t*(19) = –3.21, *p* = 0.005
EOT	12.10	2.30	11.60	3.06	12.55	1.29	*t*(11.9) = 0.91, *p* = 0.383

*^+^p < 0.10,* ^***^p < 0.001 in t-tests against 0 (see text).

Reaction times did not differ between woman and men in the current study (all *p*-values ≥0.137). Concerning questionnaire data and sex differences, significant differences between women and men were only found in the TAS-26 subscale DDF [*t*(19 = –3.21, *p* = 0.005, *d* = 1.40]. Male participants reported more difficulties describing feelings than female participants. The other comparisons did not reach significance level (all *p*-values ≥0.383).

### EEG DATA

**FIGURE 1:**
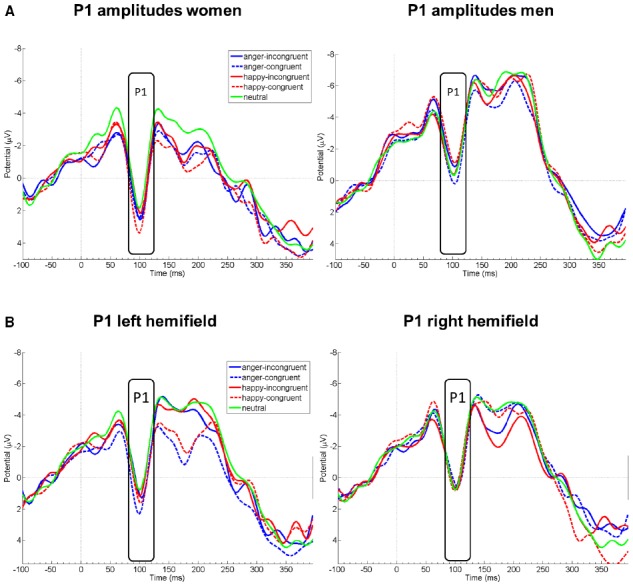
**EEG amplitude courses elicited by probe presentation at electrode Oz.** Panel **(A)** displays P1 amplitudes separately for women (left, n = 11) and men (right, n = 10). Panel **(B)** displays P1 amplitudes for all participants, separately after emotional face presentation in the left and right visual hemifield. Note that the condition neutral is included in both figures of panel **(B)** for demonstrational purposes. Negative is drawn upward per convention. The rectangles denote probe P1 analysis time window.

Probe P1 mean amplitudes differed between sexes [*F*(1,21) = 8.31, *p* = 0.009] and hemifield [*F*(1,21) = 5.94, *p* = 0.009], but not between emotions [*F*(1,20.92) = 0.93, *p* = 0.412] or congruency conditions [*F*(1,21) = 0.97, *p* = 0.397]. Moreover, there was an interaction of sex by emotion [*F*(1,21) = 3.96, *p* = 0.035]. In marginal means, women (*M* = 0.51 *μ*V, SE = 0.50) and men (*M* = –1.81 *μ*V, SE = 0.52) differed overall by a large effect size, *d* = 1.42^1^ (see Figure 1); this effect was more pronounced for happy faces (*d* = 1.76, *p* < 0.001 in simple effects analysis) than for angry (*d* = 1.14, *p* = 0.016) or neutral faces (*d* = 0.53, *p* = 0.239). The hemifields differed overall by a medium effect size, *d* = 0.65 (left: *M* = –0.31 *μ*V, SE = 0.39; right: *M* = –0.90 *μ*V, SE = 0.39).

**FIGURE 2:**
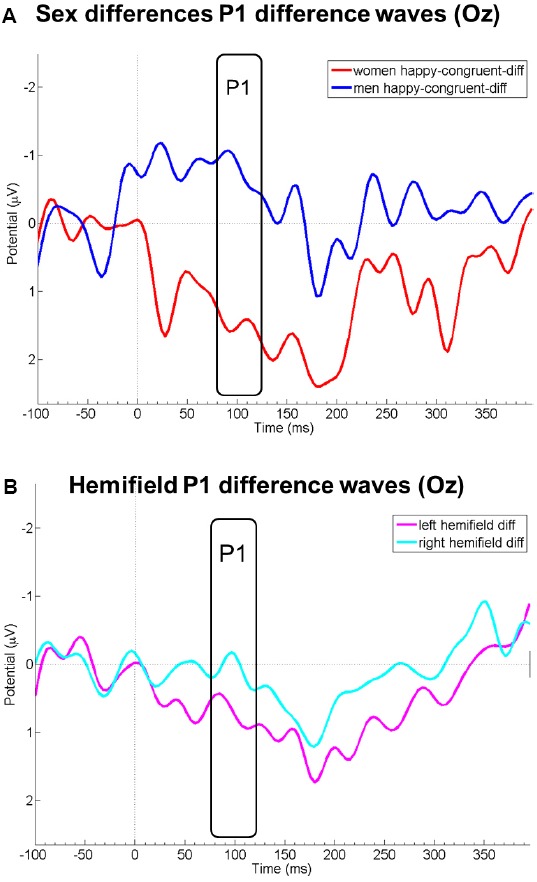
Panel **(A)** displays EEG courses at electrode Oz of the difference wave amplitudes of the condition happy-congruent minus neutral for women (red) and men (blue). Panel **(B)** displays P1 difference wave amplitudes at Oz merged for all conditions in which the emotional face was presented in the left (pink) and right (light blue) visual hemifield. Negative is drawn upward per convention. The rectangles denote probe P1 analysis time window.

Probe P1 mean difference wave amplitudes differed between right and left hemifield [*F*(1,19) = 8.08, *p* = 0.010, ${\text{η}}_{\text{p}}^{\text{2}}$ = 0.30], but not between sexes [*F*(1,19) = 1.93, *p* = 0.181, ${\text{η}}_{\text{p}}^{\text{2}}$ = 0.09], emotions [*F*(1,19) = 0.32, *p* = 0.597, ${\text{η}}_{\text{p}}^{\text{2}}$ = 0.02], or between congruency conditions [*F*(1,19) = 0.81, *p* = 0.381, ${\text{η}}_{\text{p}}^{\text{2}}$ = 0.04]. Probe P1 mean difference wave amplitudes were more positive when the emotional face was presented in the left compared to the right hemifield. All first-order interactions were not significant [sex by emotion: *F*(1,19) = 0.72, *p* = 0.407, ${\text{η}}_{\text{p}}^{\text{2}}$ = 0.04; sex by congruency: *F*(1,19) = 0.78, *p* = 0.389, ${\text{η}}_{\text{p}}^{\text{2}}$ = 0.04; sex by hemifield [*F*(1,19) = 0.25, *p* = 623, ${\text{η}}_{\text{p}}^{\text{2}}$ = 0.01; emotion by congruency: *F*(1,19) = 0.42, *p* = 0.523, ${\text{η}}_{\text{p}}^{\text{2}}$ = 0.02; emotion by hemifield: *F*(1,19) = 0.43, *p* = 0.522, ${\text{η}}_{\text{p}}^{\text{2}}$ = 0.02; congruency by hemifield: *F*(1,19) = 1.15, *p* = 0.298, ${\text{η}}_{\text{p}}^{\text{2}}$ = 0.06]. However, the triple interaction sex by emotion by congruency yielded a significant result [*F*(1,19) = 4.66, *p* = 0.044, ${\text{η}}_{\text{p}}^{\text{2}}$ = 0.20]. This could be traced to a significant difference between men and women in the *happy-congruent-diff* conditions [merged for both hemifields; *t*(19) = 2.42, *p* = 0.026, *d* = 1.06; see Figure 2]; probe P1 mean amplitudes in this condition were enhanced among women, but diminished among men relative to the *neutral* condition (see also Figure 1). This resulted in relatively higher amplitudes (i.e., more positive amplitudes) of the difference wave among women than men in the P1 time range. Men and women did not differ significantly in any of the other merged conditions (*anger-incongruent-diff*: *t*(19) = 1.32, *p* = 0.204, *d* = 0.58; *anger-congruent-diff*: *t*(19) = 0.47, *p* = 0.645, *d* = 0.21; *happy-incongruent-diff*: *t*(19) = 0.21, *p* = 0.834, *d* = 0.09). The remaining triple and the four-way interaction were not significant (all *p*-values ≥0.217).

We observed no significant correlations between bias indices and ERP amplitudes following angry (all *p*-values ≥0.074) or happy faces (all *p*-values ≥0.127) in the total sample. Separately for women and men, no significant correlations were found in women (all *p*-values ≥0.096). However, in men significant correlations were observed for DI happy scores and probe P1 mean amplitudes in the conditions *anger-congruent-R* (*r*_s_ = –0.70, *p* = 0.025) and *happy-incongruent-R* (*r*_s_ = –0.72, *p* = 0.019). The correlation between DI happy scores and probe P1 mean amplitudes of *anger-congruent-R* revealed the only significant difference between both sexes in these correlational analyses (*z* = 1.99, *p* = 0.048). Men showed a negative correlation whereas woman a non-significant positive one (*r*_s_ = 0.16, *p* = 0.631).

### ALEXITHYMIA ANALYSES

The TAS-26 subscales did not significantly inter-correlate (all *p*-values ≥0.236). Anger BI scores correlated borderline significantly with TAS-26-DDF scores (*r*_s_ = 0.42, *p* = 0.056; all other *p*-values ≥0.116). Probe P1 mean amplitudes correlated negatively with TAS-26-DIF scores in the following conditions:* anger-congruent-L* (*r*_s_ = –0.49, *p* = 0.025), *happy-incongruent-R* (*r*_s_ = –0.45, *p* = 0.041), and *happy-congruent-L* (*r*_s_ = –0.53, *p* = 0.013). Negative correlations between probe P1 mean amplitudes and TAS-26-DDF scores were observed in the *happy-congruent-L* (*r*_s_ = –0.44, *p* = 0.046) and *happy-congruent-R* conditions (*r*_s_ = –0.56, *p* = 0.008). Neither of the associations with TAS-26-DIF scores were fully qualified by participant sex (controlling for sex, the partial Spearman correlation coefficients were *r*_s_ = –0.57, *p* = 0.008; *r*_s_ = –0.49, *p* = 0.026; *r*_s_ = –0.64, *p* < 0.001), nor was the negative association of TAS-26-DDF scores with amplitudes in the *happy-congruent-R* condition (partial *r*_s_ = –0.61, *p* = 0.002); however, the association of TAS-26-DDF scores with amplitudes in the *happy-congruent-L* condition lost its significance (partial *r*_s_ = –0.31, *p* = 0.180), even though still pointing in the same direction. Probe P1 difference wave amplitudes correlated negatively with TAS-26-DDF in *happy-congruent-R-diff* (*r*_s_ = –0.44, *p* = 0.045). However, controlling for sex, this correlation was substantially reduced in magnitude and rendered insignificant, partial *r*_s_ = –0.28, *p* = 0.233.

Calculating the correlations separately in women and men, correlations with TAS-26-DIF scores remained mostly significant. In women, significant correlations were observed for probe P1 mean amplitudes for the conditions *anger-congruent-L* (*r*_s_ = –0.66, *p* = 0.027), *happy-incongruent-R* (*r*_s_ = –0.62, *p* = 0.043), and *happy-congruent-L* (*r*_s_ = –0.82, *p* = 0.002); in men only for *happy-incongruent-L* (*r*_s_ = –0.67, *p* = 0.033). The correlations between TAS-26-DIF scores and probe P1 difference wave scores yielded only a significant correlation in women for the condition *happy-congruent-L-diff* (*r*_s_ = –0.71, *p* = 0.015). No significant correlations were found for TAS-26-DDF scores and any ERPs (all other *p*-values ≥0.068). Spearman correlations between TAS-26-DIF scores and probe P1 mean amplitudes and difference wave amplitudes did not significantly differ between women and men (all *p*-values ≥0.095), neither did the correlations between TAS-26-DDF scores and the ERPs (all *p*-values ≥0.131).

## DISCUSSION

This study examined sex differences in probe P1 amplitudes, using the dot probe paradigm, and explored the usefulness of a difference wave approach for investigating attentional biases. We found that probe P1 amplitudes were overall enhanced among women, compared to men, in particular after rewarding facial stimuli. This adds to prior evidence, suggesting that neural activity during early visual processing stages is enhanced in women compared to men (Sass et al., 2010). It further underlines that neuroscientific studies may need to control and adjust for participant sex both with regard to study design and analysis (see Cahill, 2006). Moreover, we found that probe P1 amplitudes were enhanced when the emotional face was presented beforehand in the left compared to the right visual hemifield which might be due to component overlap with offset potentials of the preceding emotional stimuli. Only a few previous studies considered the factor hemifield in their analyses. Some studies observed no significant influence of hemifield (Pourtois et al., 2004; Eldar et al., 2010). However, in line with the current results, Brosch et al. (2011) observed larger probe P1 amplitudes when the probes were presented in the left hemifield. Our results can further be related to early research on hemispheric control of spatial attention. Kinsbourne (1974) postulated neuronal control networks in both hemispheres which interact in a mutually inhibitory way. Subsequent research showed that activation in one hemisphere led to orienting attention to the side of the other hemisphere (Reuter-Lorenz et al., 1990). Moreover, the bias of the right hemisphere executed on left-hemispheric activations was observed to be stronger than vice versa (Reuter-Lorenz et al., 1990). More recent theoretical assumptions emphasize competitive interactions between the hemispheres controlling spatial attention though (Szczepanski and Kastner, 2013). In any case, the observed effects of hemifield on probe P1 amplitudes further indicate hemispheric lateralization during stimulus processing in the dot probe task. Future studies should by default include hemifield in their analyses to allow stronger testing of hypotheses concerning lateralized emotional stimulus processing and spatial attention effects.

Using neutral–neutral stimulus pairs in the dot probe task, which served as a baseline in a difference wave approach, we further obtained additional preliminary evidence that, relative to this baseline, women showed specifically enhanced probe P1 amplitudes with regard to rewarding (i.e., happy) facial stimuli after congruent stimulus presentation. Previous behavioral research has suggested that healthy women show delayed disengagement specifically from happy faces (Tran et al., 2013). This could not be confirmed with the behavioral data in the present study. However, our results corroborate previous findings on the neuronal level. It may be speculated that this effect is more readily observable on a neuronal level, but demands larger sample sizes to also be observed on the behavioral level (Tran et al., 2013, investigated the data of 173 women and 174 men). Differences between men and women in the allocation of attention toward rewarding and threatening stimuli need to be investigated in much more detail in the future. Our results suggest that a difference wave approach might be optimally suited for such an endeavor and should therefore be followed up.

Using the classical mean amplitudes approach, no threat-related probe P1 amplitude variation in response to target processing was observed. This is in line with a study by Eldar et al. (2010), but is contradictory to others (Santesso et al., 2008; Brosch et al., 2011). As suggested by Eldar et al. (2010), however, different results may have been partially caused by differences in the experimental paradigms and set-ups. Task demands such as giving a motor response or withholding a motor response might have top-down influence on early stimulus processing ERPs as a function of attentional load (Fu et al., 2010). Even the temporal distance (i.e., stimulus onset asynchrony) between face pair onset and probe onset may be important, both on the behavioral (e.g., Brosch et al., 2011) and the neuronal level (Wykowska and Schubö, 2010).

The difference wave approach utilized in the present study revealed additional information which could not be captured with the classical analysis approach. The significant differences between men and women during the probe P1 time range following congruently primed happy faces suggests that women allocated more attentional resources to the happy face stimuli than men, and that their attention was still captured by these stimuli during probe presentation. In Salemink et al.’s (2007) notion, this might be interpreted as delayed disengagement from these stimuli. Emotional facial displays serve as social cues containing important information during social exchange situations (Rolls, 2000). Happy faces can be seen as approach signals initiating affiliative tendencies, whereas angry faces signal rejection and non-affiliation (Frijda et al., 1989; Bourgeois and Hess, 2008). It might be an evolutionary residue that women allocate more attentional resources to happy faces than men, thereby increasing their chances of affiliation. However, there is also ample evidence showing that gender role promotes specifically prosocial and supportive behavior in close relationships among women (Eagly, 2009), and that sociocultural influences and gender role socialization also contribute to sex differences in anxiety (McLean and Anderson, 2009).

Lastly, probe P1 and probe P1 difference wave amplitudes showed associations with self-reported difficulties in identifying and describing feelings. Participants with problems in the identification of feelings showed less activation after angry and happy faces, whereas participants with problems in describing feelings showed less activation only after happy faces in the congruent conditions. In particular women showed a negative association between difficulties in describing feelings and neuronal correlates after congruently presented happy facial displays—again in the one condition were sex differences were most evident. Previous research has suggested that alexithymia is not associated with a lack of emotional awareness *per se*, but may entail more effort in the processing of emotional information (Franz et al., 2004). Our results do not directly lend to this interpretation, but were instead suggestive of early processing deficits in alexithymia (e.g., Eichmann et al., 2008; Reker et al., 2010; Pollatos and Gramann, 2011). Applying a passive viewing paradigm, Pollatos and Gramann (2011) also observed reduced P1 amplitudes in response to emotional stimuli in participants with high levels of alexithymia, most pronounced for pleasant and neutral stimuli. Further in line with our results, these authors reported that in particular difficulties in describing feelings explained the variance in P1 amplitudes in their sample. In conclusion, our findings corroborate that alexithymia is a correlate of reduced emotional reactivity on a neuronal level. These findings need to be followed up in future studies.

Limitations pertain to the relatively small sample size and the mostly non-clinical nature of our sample which was not assessed via a structured clinical interview but via self-report. The previously reported delayed disengagement from happy faces among healthy women on the behavioral level could not be confirmed in the present study, which could, however, also be sample-related. Generally, our present findings need to be replicated in larger samples and should be, therefore, regarded only as preliminary. Moreover, sex differences in (probe) P1 amplitudes need to be investigated also in clinical samples to determine the generalizability of our results also with regard to clinical populations. The internal consistency of the externally oriented thinking factor in the TAS-26 was unacceptably low. It may not be ruled out that probe P1 amplitudes correlate also with this factor in more heterogeneous or more alexithymic samples, where a higher internal consistency of this measure may be expected.

In conclusion, the current results demonstrate that even healthy men and women differ in their neural activation while allocating attention to emotional stimuli, in particular to rewarding ones. This needs to be considered in studies of attentional biases, both in samples from the general population, but also in clinical samples. The current study was the first to use a difference wave approach to investigate attentional processes of orientation and disengagement which allowed us to detect more subtle differences between the two sexes. We recommend that future attentional bias studies include neutral–neutral trials to be able to use the difference wave approach for their research questions. Furthermore, alexithymia may need to be considered more closely in studies on attentional biases with facial stimuli.

### Conflict of Interest Statement

The authors declare that the research was conducted in the absence of any commercial or financial relationships that could be construed as a potential conflict of interest.

## Footnotes

1 Excluding the one male participant who reported elevated levels in anxiety, depression, and global psychological distress (see Participants), did not substantially change results [*F*(1,20) = 9.01, *p* = 0.007; marginal means: women: *M* = 0.49 μV, SE = 0.50; men: *M* = -1.91 μV, SE = 0.55; *d* = 1.49].

## References

[B1] Bar-Haim Y. Lamy D. Pergamin L. Bakermans-Kranenburg M. J. van Ijzendoorn M. H. (2007). Threat-related attentional bias in anxious and nonanxious individuals: a meta-analytic study. Psychol. Bull. 133, 1–24. 10.1037/0033-2909.133.1.1 17201568

[B2] Bauer H. Lauber W. (1979). Operant conditioning of brain steady potential shifts in man. Biofeedback Self Regul. 4, 145–154. 10.1007/BF01007109 476190

[B3] Berthoz S. Artiges E. Van de Moortele P.-F. Poline J. B. Rouquette S. Consoli S. M. (2002). Effect of impaired recognition and expression of emotions on frontocingulate cortices: an fMRI study of men with alexithymia. Am. J. Psychiatry 159, 961–967. 10.1176/appi.ajp.159.6.961 12042184

[B4] Blanchette I. (2006). Snakes, spiders, guns, and syringes: how specific are evolutionary constraints on the detection of threatening stimuli? Q. J. Exp. Psychol. 59, 1484–1504. 10.1080/02724980543000204 16846972

[B5] Bourgeois P. Hess U. (2008). The impact of social context on mimicry. Biol. Psychol. 77, 343–352. 10.1016/j.biopsycho.2007.11.008 18164534

[B6] Brosch T. Pourtois G. Sander D. Vuilleumier P. (2011). Additive effects of emotional, endogenous, and exogenous attention: behavioral and electrophysiological evidence. Neuropsychologia 49, 1779–1787. 10.1016/j.neuropsychologia.2011.02.056 21382388

[B7] Brown C. El-Deredy W. Blanchette I. (2010). Attentional modulation of visual-evoked potentials by threat: investigating the effect of evolutionary relevance. Brain Cogn. 74, 281–287. 10.1016/j.bandc.2010.08.008 20888109

[B8] Cahill L. (2006). Why sex matters for neuroscience. Nat. Rev. Neurosci. 7, 477–484. 10.1038/nrn1909 16688123

[B9] Cisler J. M. Koster E. H. W. (2010). Mechanisms of attentional biases toward threat in anxiety disorders: an integrative review. Clin. Psychol. Rev. 30, 203–216. 10.1016/j.cpr.2009.11.003 20005616 PMC2814889

[B10] Delorme A. Makeig S. (2004). EEGLAB: an open source toolbox for analysis of single trial EEG dynamics including independent component analysis. J. Neurosci. Methods 134, 9–21. 10.1016/j.jneumeth.2003.10.009 15102499

[B11] Derntl B. Habel U. Windischberger C. Robinson S. Kryspin-Exner I. Gur R. C. (2009). General and specific responsiveness of the amygdala during explicit emotion recognition in femals and males. BMC Neurosci. 10:91. 10.1186/1471-2202-10-91 19653893 PMC2728725

[B12] Donges U.-S. Kersting A. Suslow T. (2012). Women’s greater ability to perceive happy facial emotion automatically: gender differences in affective priming. PLoS ONE 7:e41745. 10.1371/journal.pone.0041745 22844519 PMC3402412

[B13] Eagly A. H. (2009). The his and hers of prosocial behavior: an examination of the social psychology of gender. Am. Psychol. 64, 644–658. 10.1037/0003-066X.64.8.644 19899859

[B14] Ebner N. C. Riediger M. Lindenberger U. (2010). FACES—A database of facial expressions in young, middle-aged, and older women and men: development and validation. Behav. Res. Methods 42, 351–362. 10.3758/BRM.42.1.351 20160315

[B15] Eichmann M. Kugel H. Suslow T. (2008). Difficulty identifying feelings and automatic activation in the fusiform gyrus in response to facial emotion. Percept. Mot. Skills 107, 915–922. 10.2466/pms.107.3.915-922 19235420

[B16] Ekman P. (1992). An argument for basic emotions. Cogn. Emot. 6, 169–200.

[B17] Eldar S. Yankelevitch R. Lamy D. Bar-Haim Y. (2010). Enhanced neural reactivity and selective attention to threat in anxiety. Biol. Psychol. 85, 252–257. 10.1016/j.biopsycho.2010.07.010 20655976

[B18] Franke G. H. (2002). SCL-90-R: Symptom-Checkliste von L. R. Derogatis. Göttingen: Beltz.

[B19] Franz M. Schaefer R. Schneider C. Sitte W. Bachor J. (2004). Visual event-related potentials in subjects with alexithymia: modified processing of emotional aversive information? Am. J. Psychiatry 161, 728–735. 10.1176/appi.ajp.161.4.728 15056520

[B20] Frewen P. A. Dozois D. J. A. Joanisse M. F. Nefeld R. W. J. (2008). Selective attention to threat versus reward: meta-analysis and neural-network modeling of the dot-probe task. Clin. Psychol. Rev. 28, 307–337. 10.1016/j.cpr.2007.05.006 17618023

[B21] Frijda N. H. Kuipers P. ter Schure E. (1989). Relations among emotion, appraisal, and emotional action readiness. J. Pers. Soc. Psychol. 57, 212–228 10.1037/0022-3514.57.2.212

[B22] Fu S. Fedota J. R. Greenwood P. M. Parasuraman R. (2010). Dissociation of visual C1 and P1 components as a function of attentional load: an event-related potential study. Biol. Psychol. 85, 171–178. 10.1016/j.biopsycho.2010.06.008 20599467 PMC2921581

[B23] Hillyard S. A. Anllo-Vento L. (1998). Event-related brain potentials in the study of visual selective attention. Proc. Natl. Acad. Sci. U.S.A. 95, 781–787. 10.1073/pnas.95.3.781 9448241 PMC33798

[B24] Ito T. A. Larsen J. T. Smith N. K. Cacioppo J. T. (1998). Negative information weighs more heavily on the brain: the negativity bias in evaluative categorizations. J. Pers. Soc. Psychol. 75, 887–900. 10.1037/0022-3514.75.4.887 9825526

[B25] Jessimer M. Markham R. (1997). Alexithymia: a right hemisphere dysfunction specific to recognition of certain facial expressions? Brain Cogn. 34, 246–258. 10.1006/brcg.1997.0900 9220088

[B26] Kessler R. C. Berglund P. Demler O. Jin R. Merikangas K. R. Walters E. E. (2005). Lifetime prevalence and age-of-onset distributions of DSM-IV disorders in the National Comorbidity Survey Replication. Arch. Gen. Psychiatry 62, 593–602. 10.1001/archpsyc.62.6.593 15939837

[B27] Killgore W. Yurgelun-Todd D. (2001). Sex differences in amygdala activation during the perception of facial affect. Neuroreport 12, 2543–2547. 10.1097/00001756-200108080-00050 11496145

[B28] Kinsbourne M. (1974). Direction of gaze and distribution of cerebral thought processes. Neuropsychologia 12, 279–281 10.1016/0028-3932(74)90013-X 4842386

[B29] Kupfer J. Brosig B. Brähler E. (2001). Toronto-Alexithymie-Skala-26—Deutsche Version. Göttingen: Hogrefe.

[B30] Lane R. D. Sechrest L. Riedel R. Shapiro D. E. Kaszniak A. W. (2000). Pervasive emotion recognition deficit common to alexithymia and the repressive coping style. Psychosom. Med. 62, 492–501. 10.1097/00006842-200007000-00007 10949094

[B31] Linkenkaer-Hansen K. Palva J. M. Sams M. Hietanen J. K. Aronen H. J. Ilmoniemi R. J. (1998). Face-selective processing in human extrastriate cortex around 120 ms after stimulus onset revealed by magneto- and electroencephalography. Neurosci. Lett. 253, 147–150 10.1016/S0304-3940(98)00586-2 9792232

[B32] Luck S. J. (2005). An Introduction to the Event-related Technique. Cambridge: MIT Press.

[B33] Luck S. J. Ford M. A. (1998). On the role of selective attention in visual perception. Proc. Natl. Acad. Sci. U.S.A. 95, 825–830. 10.1073/pnas.95.3.825 9448247 PMC33804

[B34] MacLeod C. Mathews A. Tata P. (1986). Attentional bias in emotional disorders. J. Abnorm. Psychol. 95, 15–20. 10.1037/0021-843X.95.1.15 3700842

[B35] Mantani T. Okamoto Y. Shirao N. Okada G. Yamawaki S. (2005). Reduced activation of posterior cingulate cortex during imagery in subjects with high degrees of alexithymia: a functional magnetic resonance imaging study. Biol. Psychiatry 57, 982–990. 10.1016/j.biopsych.2005.01.047 15860338

[B36] McLean C. P. Anderson E. R. (2009). Brave men and timid women? A review of the gender differences in fear and anxiety. Clin. Psychol. Rev. 29, 495–505. 10.1016/j.cpr.2009.05.003 19541399

[B37] Öhman A. Flykt A. Esteves F. (2001). Emotion drives attention: detecting the snake in the grass. J. Exp. Psychol. Gen. 130, 466–478. 10.1037/0096-3445.130.3.466 11561921

[B38] Oldfield R. C. (1971). The assessment and analysis of handedness: the Edinburgh Inventory. Neuropsychologia 9, 97–113 10.1016/0028-3932(71)90067-4 5146491

[B39] O’Toole L. Dennis T. A. (2012). Attention training and the threat bias: an ERP study. Brain Cogn. 78, 63–73. 10.1016/j.bandc.2011.10.007 22083026 PMC3233611

[B40] Picton T. W. Hillyard S. A. (1972). Cephalic skin potentials in electroencephalography. Electroencephalogr. Clin. Neurophysiol. 33, 419–424 10.1016/0013-4694(72)90122-8 4115700

[B41] Pizzagalli D. A. Lehmann D. Hendrick A. M. Regard M. Pascual-Marqui R. D. Davidson R. J. (2002). Affective judgments of faces modulate early activity (approximately 160 ms) within the fusiform gyri. Neuroimage 16, 663–677. 10.1006/nimg.2002.1126 12169251

[B42] Pollatos O. Gramann K. (2011). Electrophysiological evidence of early processing deficits in alexithymia. Biol. Psychol. 87, 113–121. 10.1016/j.biopsycho.2011.02.016 21376102

[B43] Pourtois G. Grandjean D. Sander D. Vuilleumier P. (2004). Electrophysiological correlates of rapid spatial orienting towards fearful faces. Cereb. Cortex 14, 619–633. 10.1093/cercor/bhh023 15054077

[B44] Reker M. Ohrmann P. Rauch A. V. Kugel H. Bauer J. Dannlowski U. (2010). Individual differences in alexithymia and brain response to masked emotion faces. Cortex 46, 658–667. 10.1016/j.cortex.2009.05.008 19524887

[B45] Reuter-Lorenz P. A. Kinsbourne M. Moscovitch M. (1990). Hemispheric control of spatial attention. Brain Cogn. 12, 240–266 10.1016/0278-2626(90)90018-J 2340154

[B46] Rolls E. T. (2000). The orbitofrontal cortex and reward. Cereb. Cortex 10, 284–294. 10.1093/cercor/10.3.284 10731223

[B47] Salemink E. van den Hout M. A. Kindt M. (2007). Selective attention and threat: quick orienting versus slow disengagement and two versions of the dot probe task. Behav. Res. Ther. 45, 607–615. 10.1016/j.brat.2006.04.004 16769035

[B48] Santesso D. L. Meuret A. E. Hofmann S. G. Mueller E. M. Ratner K. G. Roesch E. B. (2008). Electrophysiological correlates of spatial orienting towards angry faces: a source localization study. Neuropsychologia 46, 1338–1348. 10.1016/j.neuropsychologia.2007.12.013 18249424 PMC2441935

[B49] Sass S. M. Heller W. Stewart J. L. Levin Silton R. Edgar J. C. Heller W. (2010). Time course of attentional bias in anxiety: emotion and gender specificity. Psychophysiology 47, 247–259. 10.1111/j.1469-8986.2009.00926.x 19863758 PMC3073148

[B50] Schaefer R. Schneider C. Tress W. Franz M. (2007). Cortical augmenting in alexithymic subjects after unpleasant acoustic stimulation. J. Psychosom. Res. 63, 357–364. 10.1016/j.jpsychores.2007.03.015 17905042

[B51] Sifneos P. E. (1976). The prevalence of ‘alexithymic’ characteristics in psychosomatic patients. Psychother. Psychosom. 22, 255–262 10.1159/000286529 4770536

[B52] Smith N. K. Cacioppom J. T. Larsen J. T. Chartrand T. L. (2003). May I have your attention, please: electrocortical responses to positive and negative stimuli. Neuropsychologia 41, 171–183 10.1016/S0028-3932(02)00147-1 12459215

[B53] Staugaard S. R. (2010). Threatening faces and social anxiety: a literature review. Clin. Psychol. Rev. 30, 669–690. 10.1016/j.cpr.2010.05.001 20554362

[B54] Stephenson W. Gibbs F. A. (1951). A balanced non-cephalic reference electrode. Electroencephalogr. Clin. Neurophysiol. 3, 237–240 10.1016/0013-4694(51)90017-X 14840404

[B55] Szczepanski S. M. Kastner S. (2013). Shifting attentional priorities: control of spatial attention through hemispheric competition. J. Neurosci. 33, 5411–5421. 10.1523/JNEUROSCI.4089-12.2013 23516306 PMC3651512

[B56] Tan J. Ma Z. Gao X. Wu Y. Fang F. (2011). Gender difference of unconscious attentional bias in high trait anxiety individuals. PLoS ONE 6:e20305. 10.1371/journal.pone.0020305 21647221 PMC3101250

[B57] Tran U. S. Lamplmayr E. Pintzinger N. M. Pfabigan D. M. (2013). Happy and angry faces: subclinical levels of anxiety are differentially related to attentional biases in men and women. J. Res. Personal. 47, 390–397 10.1016/j.jrp.2013.03.007

[B58] Vuilleumier P. Richardson M. P. Armony J. L. Driver J. Dolan R. J. (2004). Distant influences of amygdala lesion on visual cortical activation during emotional face processing. Nat. Neurosci. 7, 1271–1278. 10.1038/nn1341 15494727

[B59] Waechter S. Nelson A. L. Wright C. Hyatt A. Oakman J. (2014). Measuring attentional bias to threat: reliability of dot probe and eye movement indices. Cogn. Ther. Res. 38, 313–333 10.1007/s10608-013-9588-2

[B60] Wykowska A. Schubö A. (2010). On the temporal relation of top-down and bottom-up mechanisms during guidance of attention. J. Cogn. Neurosci. 22, 640–654. 10.1162/jocn.2009.21222 19309292

[B61] Yiend J. (2010). The effects of emotion on attention: a review of attentional processing of emotional information. Cogn. Emot. 24, 3–47 10.1080/02699930903205698

